# Primary care professionals’ views on population-based expanded carrier screening: an online focus group study

**DOI:** 10.1093/fampra/cmad011

**Published:** 2023-02-01

**Authors:** Lieke M van den Heuvel, Anke J Woudstra, Sanne van der Hout, Suze Jans, Tjerk Wiersma, Wybo Dondorp, Erwin Birnie, Phillis Lakeman, Lidewij Henneman, Mirjam Plantinga, Irene M van Langen

**Affiliations:** Department of Genetics, University Medical Centre Groningen/University of Groningen, Groningen, The Netherlands; Department of Human Genetics and Amsterdam Reproduction and Development Research Institute, Amsterdam UMC, Vrije Universiteit, Amsterdam, The Netherlands; Department of Human Genetics and Amsterdam Reproduction and Development Research Institute, Amsterdam UMC, Vrije Universiteit, Amsterdam, The Netherlands; Department of Health, Ethics & Society, Maastricht University Medical Centre/Maastricht University, Maastricht, The Netherlands; Department of Child Health, TNO, Leiden, The Netherlands; Dutch College of General Practitioners, Utrecht, The Netherlands; Department of Health, Ethics & Society, Maastricht University Medical Centre/Maastricht University, Maastricht, The Netherlands; Department of Genetics, University Medical Centre Groningen/University of Groningen, Groningen, The Netherlands; Department of Human Genetics and Amsterdam Reproduction and Development Research Institute, Amsterdam UMC, University of Amsterdam, Amsterdam, The Netherlands; Department of Human Genetics and Amsterdam Reproduction and Development Research Institute, Amsterdam UMC, Vrije Universiteit, Amsterdam, The Netherlands; Department of Genetics, University Medical Centre Groningen/University of Groningen, Groningen, The Netherlands; Department of Genetics, University Medical Centre Groningen/University of Groningen, Groningen, The Netherlands

**Keywords:** expanded carrier screening, focus groups, general practitioners, midwives, primary care professionals, views

## Abstract

**Background:**

Population-based expanded carrier screening (ECS) involves screening for multiple recessive diseases offered to all couples considering a pregnancy or during pregnancy. Previous research indicates that in some countries primary care professionals are perceived as suitable providers for ECS. However, little is known about their perspectives. We therefore aimed to explore primary care professionals’ views on population-based ECS.

**Methods:**

Four online focus groups with 14 general practitioners (GPs) and 16 community midwives were conducted in the Netherlands.

**Results:**

Our findings highlight various perspectives on the desirability of population-based ECS. Participants agreed that ECS could enhance reproductive autonomy and thereby prevent suffering of the child and/or parents. However, they also raised several ethical, societal, and psychological concerns, including a tendency towards a perfect society, stigmatization, unequal access to screening and negative psychosocial consequences. Participants believed that provision of population-based ECS would be feasible if prerequisites regarding training and reimbursement for providers would be fulfilled. most GPs considered themselves less suitable or capable of providing ECS, in contrast to midwives who did consider themselves suitable. Nevertheless, participants believed that, if implemented, ECS should be offered in primary care or by public health services rather than as hospital-based specialized care, because they believed a primary care ECS offer increases access in terms of time and location.

**Conclusions:**

While participants believed that an ECS offer would be feasible, they questioned its desirability and priority. Studies on the desirability and feasibility of population-based ECS offered in primary care or public health settings are needed.

**Key messages**

- GPs and midwives are potential providers of population-based ECS.
- Primary care professionals believed that ECS enhances reproductive autonomy.
- But they questioned desirability due to ethical, societal, and psychological concerns.
- They considered primary care a preferred setting for population-based ECS.
- It was considered feasible, if training, time, and reimbursement is guaranteed.

## Introduction

Expanded carrier screening (ECS) involves screening of couples to establish their carrier status for multiple recessive diseases.^1^ With more than 1,900 recessive diseases known,^2,3^ it is estimated that approximately 1 in 100/125 couples in the general population are at risk of conceiving a child affected with a severe recessive condition.^4^ Carrier screening allows couples to be informed about their potentially increased risk. When testing is offered preconceptionally, it enables couples with increased risk to consider a maximum of reproductive options, including accepting the risk, preimplantation genetic testing, prenatal diagnosis, refraining from having (biological) children, the use of donor gametes, and adoption. In contrast, carrier screening offered during pregnancy limits the reproductive options for carrier couples.

Until recently, carrier screening for only 1 or a few diseases was offered to couples based on ancestry. Carrier screening has now become more sensitive, faster, and affordable, allowing for an expanded offer to all couples planning to conceive that tests for multiple recessive diseases simultaneously.^1^ ECS offered to all couples with a wish for children or during pregnancy is referred to as population-based ECS.^5,6^

While pilots for population-based ECS have been carried out in several countries including the Netherlands^7,8^ and Australia,^9^ population-based ECS has not been implemented as a screening programme anywhere in the world.^10^ In the Netherlands, 2 academic hospitals developed an ECS test for 50–70 severe childhood onset disorders, which is currently available on request for couples planning a pregnancy (out-of-pocket cost). In May 2022, the Minister of Health has asked the Health Council of the Netherlands for policy advice with regard to the possible implementation of population-based ECS.

To enable population-based ECS, a considerable number of knowledgeable healthcare professionals (HCPs) will be needed to provide (pretest) counselling. However, HCPs in secondary and tertiary care, such as clinical geneticists and gynaecologists, will not be sufficiently available or easily accessible to provide this counselling. Primary care professionals could be suitable to fill this gap, and a few studies have indicated that primary care professionals, such as community midwives and general practitioners (GPs), are considered appropriate providers of population-based ECS.^11,12^ The HCPs in these studies also mentioned primary care professionals as suitable providers of ECS. Recently, a pilot study conducted in the Netherlands indicated that a couple-based ECS offer by trained and motivated GPs was considered feasible and acceptable by couples.^7^ GPs and midwives also play an important role in providing ECS in “Mackenzie’s Mission,” an Australian research programme offering ECS to 10,000 couples.

Although population-based ECS could promote reproductive autonomy for all couples with a child wish, previous literature also raised ethical, societal, and psychological concerns, including a potentially increased risk on stigmatization and discrimination,^10,13^ challenges in achieving equitable access,^14^ and distress among couples while waiting for the result or for those identified as a carrier couple.^5^ Previous research among HCPs’, including clinical geneticists, genetic counsellors, and gynaecologists, on the desirability of reproductive screening for 1 or a few diseases^15–17^ as well as on population-based ECS^7,18–24^ also indicated their concerns on these ethical, societal, and psychological issues when offering population-based ECS.

While primary care professionals are thus considered suitable providers of population-based ECS, there is little knowledge about their perspectives. Therefore, this focus group study aimed to explore the views of GPs and community midwives regarding desirability and feasibility of population-based ECS, including their and other HCP’s potential role and responsibilities in such an offer.

## Methods

### Setting

In the Netherlands, population-based carrier screening (including ECS) is not embedded in mainstream healthcare or in programmatic reproductive screening offers.^25^ Clinical geneticists or genetic counsellors may offer carrier screening opportunistically to high-risk couples based on ancestry and/or consanguinity, and this screening is covered by healthcare insurance. In addition, some primary care midwifery practices offer haemoglobinopathy carrier screening to high-risk women, and GPs are also eligible to do so.^25^ In 2 pilot studies conducted at academic hospitals licenced for genetic counselling and genome diagnostics, ECS was offered to couples from the general population, and one of these 2 studies successfully offered ECS via GPs.^7,8^ Couples can still request these tests (at cost-price), but there are no commercial ECS providers in the Netherlands as this is legally prohibited. Dutch GPs and community midwives are gatekeepers for referral to secondary and tertiary healthcare. Both may offer preconception care aimed at facilitating healthy pregnancies, but this is still very rarely requested. Most pregnant women in the Netherlands are monitored in primary midwifery care, which is fully reimbursed. However, despite extensive efforts, the delivery and uptake of preconception care are low.^26^

### Design

We chose a qualitative focus group design to reach our study objectives since this allowed us to explore different perspectives and promote discussions between participants. In this study, we followed the principles of a grounded theory approach in that we conducted our process of data collection and analysis in an iterative and comparative manner.^27^

### Participants and procedure

A convenience sampling strategy was used. Midwives and GPs were approached for participation via relevant Dutch professional networks, such as the Department of Family and Geriatric Medicine (AHON, see Supplementary Material S1 for a list of networks approached), primarily networks residing in the Northern part of the Netherlands. Also GPs and midwives who were still in training were also approached for participation. To increase interest in participation, prior to the focus groups, participants were offered an accredited training by a clinical geneticist (PL) on haemoglobinopathies and the Dutch carrier screening guideline for recessive diseases in high-risk groups and a financial incentive of €50 (for midwives) and €100 (for GPs). Snowball sampling was also used by asking those indicating interest whether their colleagues would also be interested in participating. Those interested in study participation received additional information about the study (including its aim and design) per email and were asked to confirm their consent for study participation.

### Data collection

A total of 4 focus groups were conducted. The focus groups were conducted online instead of face-to-face, because of the COVID-19 pandemic. Focus groups were organized asynchronously (with participants logging in to them at a suitable time) or synchronously (with participants being online simultaneously), according to participants’ preferences. We chose to organize both asynchronous focus groups as synchronous focus groups to optimize study accessibility for participants with limited time available and/or irregular time schedules.^28^ Two online asynchronous focus groups (1 with GPs, 1 with midwives) were conducted in 7 days in which participants received 1 or 2 questions each day, to which they were asked to respond. These groups were organized using the focusgroupit.com tool. In addition, two 1.5-h synchronous focus groups (1 with GPs, 1 with midwives) were held using Cisco Webex, and these were video recorded. The executing researcher (LMvdH), who is experienced in qualitative research, moderated the focus groups. She had no relationship with the participants prior to this focus group study and the participants did not receive any personal knowledge about the interviewer. An observer (EB or IvL) was present during each focus group making field notes. The semistructured topic list comprised the following topics: (i) views on (expanded) preconception carrier screening in general, (ii) ethical aspects and consequences of offering ECS, (iii) ECS as part of prenatal screening, and (iv) ideas regarding implementation of ECS and the potential role of different HCPs, including their own profession (Supplementary Material S2). The topic list was not piloted prior to the focus groups. Data were collected between October 2020 and February 2021.

### Data analysis

Ad verbatim transcripts were analysed using Atlas.ti software version 5.2, based on the principles of thematic content analysis^29^ and grounded theory.^27^ Coding analysis was performed across the focus groups, including both the groups with GPs and the groups with midwives. Initial coding, comprising open coding and category identification,^27^ was performed by 2 researchers independently (LMvdH and AJW), in which we analysed the first 2 focus groups to guide the analysis of the second 2 focus groups. Subsequently, a codebook was developed and discussed with the research group. Based on the codebook, main themes and subthemes were derived. Transcripts were re-read repeatedly during the process by LMvdH to make sure no relevant findings were missed and to check whether the main themes and subthemes were consistent with the data.

## Results

### Participants

In total, 16 GPs and 16 midwives indicated interest, of whom 14 GPs and 16 midwives eventually participated. Table 1 shows participants’ characteristics. All the midwives and half of the GPs were female. 5/14 GPs and 7/16 midwives were still in training, all being experienced in patient care. Just more than half (17/30) resided in the northern part of the Netherlands; 9/14 GPs and 8/16 midwives worked rurally, while the others worked in a city. Half of the midwives had experience in offering preconception care, as compared with just 2 of the GPs, who had participated in the earlier ECS pilot^7^ and were still offering ECS.

**Table 1.** Sociodemographic and professional characteristics of participants.

Characteristic	GPs	Midwives
	*N* (%)	*N* (%)
Gender		
Male	7 (50.0)	0 (0.0)
Female	7 (50.0)	16 (100.0)
Years of work experience		
In training	5 (35.7)	7 (43.8)
1–10 years	4 (28.6)	3 (18.8)
11–30 years	4 (28.5)	4 (25.1)
>30 years	1 (7.2)	2 (12.5)
Practice region		
Northern Netherlands	10 (71.4)	7 (43.8)
Western Netherlands	0 (0.0)	6 (37.5)
Southern Netherlands	1 (7.1)	0 (0.0)
Eastern Netherlands	2 (14.3)	1 (6.3)
Middle of the Netherlands	1 (7.1)	2 (12.5)
Practice type		
City	5 (35.7)	8 (50.0)
Rural	9 (64.3)	6 (37.5)
Unknown	0 (0.0)	2 (12.5)
Number of patients in GP practice		
1,500 to <2,500 patients	5 (3.6)	
2,500 to <3,500 patients	6 (42.9)	
≥3,500 patients	3 (10.3)	
Number of patients in midwifery practice		
100 to <200 patients		6 (40.0)
200 to <300 patients		2 (13.3)
300 to <400 patients		3 (20.0)
≥400 patients		5 (33.3)
Provision of preconception care by participant		
Yes	2 (14.3)	7 (43.8)
No	12 (85.7)	9 (56.3)

### Themes

Table 2 shows the main themes and subthemes identified, with illustrative quotes per theme.

**Table 2.** Illustrative quotation per theme and subthemes.

No.	Profession	Quotation
*Aim of population-based ECS*		
1	Midwife	The purpose of population-based ECS is to offer (future) parents’ options for action, whereby I realise that by naming the choices as action options, the emphasis is on action, while the choice of waiting/preparing/refraining from having a (biological) child may be overshadowed (P3, FG2).
2	Midwife	For me, the aim would be to prevent suffering. I think it is better that future parents are enabled to make an informed decision instead of being unaware until birth (P7, FG2).
3	Midwife in training	I agree that we have to look at how to reduce healthcare costs, but I think it is a very radical choice to offer it from that point of view, because then you are implicitly saying to parents that we as a society believe that your child should not exist if it has [such] a condition so you are going to influence decision-making of [future] parents regarding carrier screening (P2, FG1).
*Desirability of population-based ECS*		
4	GP	I think that in terms of insurance it does not belong in the basic package, because then the premium rises and it may be at the expense of other—more urgent—choices in healthcare. But that is open to discussion. I don’t think it should become a standard offer for all couples wishing to have children (P5, FG3).
5	Midwife in training	I find it difficult eh … Yes, I wonder from which point of view one may say that it’s a luxury thing, because I wonder how for example parents who were not necessarily at high risk who find out during pregnancy that their child is affected and that woman has to give birth at 26 weeks. I wonder if they think it’s a luxury. I think they would very much have liked to know that in advance to avoid that suffering. So it’s only a luxury for the people who do have healthy children (P6, FG1).
*Ethical, societal, and psychosocial concerns*		
6	GP	Medicalisation in itself is not necessarily wrong. Preventing disease prevents suffering (and saves money) in our bulging world civilisation), which can be positive. However, an engineered society is its downside. … It suggests that we have to live in a perfect world, where no “mistakes” are allowed, while many people with disabilities and diseases might as well be happy (P8, FG3).
7	GP	I think that when a child has a heritable or chromosomal disease, in one way or another there is always the tendency from society or child or parent themselves to look at someone for that, because we have tests that could have predicted that. I think this can also be a problem for the individual: Accusations towards the parents perhaps?… Perhaps even healthcare professionals will have an opinion on this, which may even have an unintended effect on the care (extra eyes on the parents). It is impossible to predict how this will develop, but unfortunately, we humans are always inclined to point the finger of blame when something goes wrong, especially when it could have been prevented (P8, FG3).
8	Midwife	There is pressure from society, I hear so often in the consulting room what they must choose from their mother, mother-in-law, neighbour, aunt, etc., because “don’t be so weird, we all did it too” to “no yuck, the last thing you want for your child is a cleft lip and palate”… Then try to choose for yourself if you think differently (P2, FG2).
9	Midwife	I think that, particularly, this subject is difficult for couples or pregnant women with a lower level of education and [they] have difficulty interpreting and weighing the information. In advance, this group (who may indeed more often become pregnant unplanned) should not be deprived of it, because they missed the opportunity. So it should also be done at the beginning of the pregnancy, before a certain period (depending on how quickly screening and any subsequent diagnostics can be done) (P2, FG1).
10	Midwife	It [population-based ECS] creates opportunities but also a lot of uncertainty, a lot of distress about having to choose, getting along with your partner, having to deal with the opinions of your social circle—“Do you have a child with cystic fibrosis? Did you not get tested?”—and the guilt that may come with that. It’s tough, though, because when you’re dealing with a child with a disability or even death, and it technically could have been sorted out in advance, with the choices involved, I can imagine people saying, “Why wasn’t I told? Why didn’t I have a choice?” (P10, FG2).
*Feasibility of ECS*		
11	Midwife	Young people could be generally informed, so that they can form their own opinion on the topic. Becoming pregnant is something you do together, so then [later on] two already somewhat informed young adults hopefully come to a jointly supported decision when it [having children] is actually on the agenda (P6, FG2).
*Providers of population-based ECS*		
12	GP	Well, I’ve seen how it goes with the prenatal test [NIPT] and I see that there… I have also personally experienced that the counselling was zero and that we were just offered the test and she was told “the woman is over thirty-five, so a neck fold measurement is required.” That was zero counselling, so I don’t really have any confidence that they [midwives] have enough time and attention for that to be done properly (P3, FG4).
13	GP	I disagree with [participant name]. Expertise can be collected; you can choose to make time for it. In recent years, more and more tasks are assigned to the GP. I think that this is often the reason for the reluctant attitude: “Another thing, and I already have so little time.” However, I think that we as GPs can also change our profession, we can delegate more and more, employ back-office assistants, a physician assistant—you name it. This would allow GPs to make time for these more complex patient questions (P9, FG3).
14	Midwife in training	Yes, I think that would be a bit unfair if you would only offer it to people who come in with the question, because then I can imagine that you would pick out the highly educated people among them…. So no, I don’t think you should offer it to people who ask for it themselves (P3, FG1).

FG, focus group; P, participant.

#### Perceived aim of population-based ECS

Many participants perceived 2 aims of a population-based ECS offer: (i) to enhance reproductive autonomy and (ii) to prevent suffering of the child and/or the parents. Regarding reproductive autonomy, most participants considered the possibility of informing couples in a timely manner about their risk of conceiving an affected child and the preventive options important. One of the participating midwives related enhancing autonomy to couples’ desire to “know everything.” However, it was also stated that the aim of enhancing reproductive autonomy may give couples the idea that they are expected to act upon the information they received (Quote 1, Table 2).

Regarding prevention of suffering, ECS was said to prevent suffering of both prospective parents and children by enabling future parents to make informed choices, instead of being unaware of their risk until confronted with the birth of an affected child (Quote 2, Table 2). Some participants explained that their focus on the aim of prevention was linked to their own experience with the impact of serious genetic diseases. It was also mentioned that suffering can be perceived differently for similar diseases. Participants differed in their opinion on what prevents suffering most. All participants agreed that ECS should focus on serious, early-onset diseases that significantly limit life expectancy and for which no treatment is available. Furthermore, they shared other ideas on diseases screened for, including diseases heavily affecting quality of life, diseases requiring treatment very early in life to prevent neonatal death and serious later-onset diseases where early knowledge would enable timely adaptation to the future. Some participants also indicated that the decision of what ECS should screen for should be restricted and clear in order to prevent a slippery slope. One GP also referred to the right to know health information as a reason to implement population-based ECS. Some participants mentioned that decreasing healthcare costs could also be a consequence of population-based ECS, but they clearly stated that this should not be considered a primary aim, because it would contribute to the idea that there is no place for affected children in our society (Quote 3, Table 2).

#### Desirability of population-based ECS

About half of participants, more GPs than midwives, preferred the already existing high-risk (ancestry-based) carrier screening offer to population-based ECS. This was related to perceived prevalence of and experiences with the diseases screened for in these respective categories. Some perceived the cumulative prevalence of serious recessive diseases and the risk of having an affected child as high, others as low. A few midwives considered population-based ECS to be part of a development that cannot be stopped, regardless of its desirability. Some participants also considered a population-based offer undesirable because they questioned couples’ ability to adequately decide about screening and their ability to comprehend its consequences. Other participants questioned the desirability of a government- or healthcare-based offer but regarded a commercial offer as even less desirable. For some participants, population-based ECS was not considered a priority because of the rarity of these diseases and believed it should not be offered or that couples should pay for it (Quote 4, Table 2). Others, however, believed it to be important, considering that most parents are unaware of their risks or of the severity of the diseases ECS screens for and the reproductive options available (Quote 5, Table 2).

#### Ethical, societal, and psychosocial concerns

##### Sliding towards a “perfect society”

Concerns regarding an undesirable increase of “medicalisation” surrounding reproduction were raised. Participants stated that medicalisation is not per se undesirable but found it undesirable when medicalisation results in routinization and societal pressure or a push towards a “perfect society” (Quote 6, Table 2).

##### Stigmatization and discrimination

Participants feared stigmatization of carriers and/or affected children and parents. Some also believed that population-based ECS (and high uptake, leading to lower prevalence of the diseases in the test) may limit the possibilities to fund research on treatment options for the diseases screened for. One GP also suggested that it may lead to stigmatization by HCPs and may even affect HCPs in the care they provide for these children (Quote 7, Table 2). Related to concerns about stigmatization, many participants, both GPs and midwives, feared that individuals or couples will experience societal pressure to partake. Some midwives already experienced societal pressure on their clients in decision-making about prenatal screening (Quote 8, Table 2).

##### Concerns regarding equal access

All GPs and midwives worried about how to ensure equal access to screening. Many participants were concerned that access to screening might be limited by poor understanding of and awareness about the screening offer, costs and language barriers (Quote 9, Table 2).

##### Psychological impact of population-based ECS

Some participants shared concerns about the psychological impact of population-based ECS on couples. Many participants believed that population-based ECS could lead to uncertainty and distress related to decision-making, as well as impact partner relationships if partners cope differently with the decision. On the other hand, some participants reported that the current generation of couples of reproductive age is more familiar with decisions about health and screening, which may limit negative psychosocial consequences. One midwife in training wondered whether the potentially high psychosocial impact on couples is a valid reason for deciding against population-based ECS, specifically in the case of a preconceptional offer, considering the profound psychological impact of having a child affected by a serious condition (Quote 10, Table 2).

#### Feasibility of population-based ECS

Many participants considered a population-based ECS offer in itself feasible, although several requirements should be met, such as the need for (i) adequate pretest genetic counselling, (ii) raised awareness of population-based ECS to promote equal access, and (iii) financial reimbursement and training for providers of population-based ECS. Participants agreed that, if implemented, population-based ECS should primarily be offered preconceptionally, as this allows couples the widest range of reproductive options. Others also believed that reproductive options restricted to the preconception phase, such as preimplantation genetic testing, are morally more acceptable than the options available during pregnancy. Many participants thought it would be ideal to offer ECS as part of existing preconception care consultations, as they believed this might increase the uptake of preconception care as an additional benefit. While a preconception offer was thought to be preferable, participants also mentioned that unplanned pregnancies are more common among couples with low socioeconomic status, and therefore these participants believed an exclusively preconceptional offer would be limiting and could contribute to unequal access. Participants therefore felt a combined preconception/prenatal offer would be optimal. Some participants argued for differentiated timing in offering information about preconception ECS and offering pretest counselling: general information to raise awareness could be provided to young people during high school, for example, while the actual offer could be provided when there is an active wish to conceive (Quote 11, Table 2).

#### Providers of population-based ECS

Almost all participants supported the idea that population-based ECS should be provided in primary care or by public health services, as this would be easily accessible for couples. Most midwives considered themselves appropriate providers of population-based ECS. Their experience with prenatal screening made them feel capable of providing ECS counselling. Many GPs also considered midwives suitable. Participants believed ECS is part of maternity care, and it therefore makes sense that midwives are the preferred provider, especially as part of preconception care. Moreover, ECS provision by midwives could contribute to continuity of care when a woman later becomes pregnant. One GP however questioned the time available and knowledge of midwives (Quote 12, Table 2).

In contrast to the midwives, many GPs did not want to provide population-based ECS. Whilst they believed ECS should be offered by an easily accessible and trusted provider, many of them perceived barriers, including lack of time, lack of expertise, and little contact with couples who wish to have children. They also stated that they work using a problem-driven approach and that they perceived prevention (such as ECS, in their opinion) as not their professional task. However, some GPs believed that the barriers previously mentioned can be solved by expanding resources, for example by employing physician assistants (Quote 13, Table 2). Other providers suggested were fertility specialists and gynaecologists. Some believed that certain professionals from these disciplines should specialize in ECS. Others believed this should not be the case because they thought this could decrease accessibility to ECS. All participants agreed that providers should be trained in pretest counselling.

In addition, if population-based ECS would be implemented, participants believed that the government should organize active, low cost, systematic provision of ECS to promote equal access to screening and limit participation in commercial screening. It was considered important that, if the government were to organize population-based ECS, couples should not be encouraged to partake in screening comparable with how prenatal screening offers have been implemented emphasizing couples’ freedom to choose. Some participants also believed that ECS organized by the government may give people the idea that partaking in this kind of screening is the right thing to do. Others however felt that it should be actively offered to allow every interested couple to partake in ECS (Quote 14, Table 2).

## Discussion

This focus group study exposed various views on the desirability of population-based ECS. Despite differences in the perceived desirability of ECS, most considered a population-based ECS offer in itself feasible, once certain requirements are met. The requirements for implementation mentioned were similar to the requirements raised in a previous study among Dutch stakeholders, outlined on “Culture” (e.g. desirability and prioritization) “Structure” (e.g. counselling, education and training, reimbursement) and “Practice” (e.g. responsibility for implementation) level.^30^

The perceived desirability of population-based ECS among participants was based on ethical, legal, and societal issues, and seemed to be related, at least in part, to how participants perceive the risk of being a carrier couple and/or of conceiving an affected child, which was also found in previous research on genetic and nongenetic HCPs’ views on population-based ECS.^21,30^ Although professionals raised concerns about ethical, legal, and psychosocial issues, including a tendency towards a perfect society, stigmatization, and unequal access, empirical evidence is lacking.^14^ Multiple participants questioned couples’ capacity to decide about partaking in screening because they thought many couples could not comprehend the consequences; others, both GPs and midwives, however believed that all couples have a right to be offered ECS despite potential challenges for informed decision-making. This was also mentioned by stakeholders in the study of van der Hout et al.,^31^ who stated that it would be paternalistic and unethical to deprive people of such information and testing. Participants in this study stressed the importance of adequate counselling of couples by skilled providers to promote informed decision-making, a view also shared by other stakeholders.^32,33^

Literature on reproductive screening reveals broad consensus that the aim of carrier screening should be to enhance reproductive autonomy by giving parents the option of avoiding suffering for the child, themselves and/or their family, rather than to contribute to prevention in the sense of achieving population-level health gains.^31^ Multiple GPs and midwives in our study stated that the aim of population-based ECS should not only be enhancing reproductive autonomy, but also the prevention of suffering of children and/or their parents. This idea of prevention of suffering as something to be achieved by the programme was particularly stated by participants who had professional or personal experience with children severely affected by such a condition. Whereas in the ethical literature autonomy and prevention (in the sense of achieving population-level health gains) are often regarded as fundamentally opposing aims,^31^ these participants seemed not to share this understanding. As this may influence pretest counselling, it is important to further explore what potential providers perceive as the aim(s) of ECS, also considering their opinions on parental and professional responsibilities.

Multiple participants stated that ECS should be offered not only preconceptionally but also prenatally, as this would promote equal access to screening. This was also found in another study interviewing HCPs.^21^ However, preconception ECS was considered preferable because it enables a wider range of reproductive options, but also because it fits in with already existing (but still underused) offers of preconception care, confirming the views expressed in the review of Best et al.^11^ However, it has been argued that, given that current preconception care is provided from a prevention perspective (advising potential mothers to quit alcohol and smoking and take folic acid), using this as a setting for offering carrier screening may be at odds with the ideal of nondirective counselling.^34^ Whereas some degree of directive counselling may well be acceptable for lifestyle advice, it would decidedly not be appropriate for carrier screening in which a nondirective, decisional counselling is preferred. Also after testing, if a carrier couple is identified, nondirective counselling by genetic professionals in which psychological assistance is provided to support couple’s reproductive decision-making, is strongly recommended.^5^ It is at least important that providers are aware of these different aims and adapt their counselling-style accordingly.

In this study, most GPs considered themselves less suitable as providers of population-based ECS. The barriers they perceived are comparable to barriers perceived by GPs in previous research.^11^ In addition, these GPs believed it is not a GP’s task to offer ECS, because they did not perceived it as their role to promote prevention. Interestingly, previous research indicates that members of the general population perceived GPs as a suitable and trusted provider of population-based ECS.^9,12,35^ A solution would be for certain GPs or midwives who are interested in ECS to specialize in offering it, as was indicated in our focus groups. Moreover, pilot studies in the Netherlands indicated that motivated, trained GPs are able to appropriately provide carrier screening for single diseases (cystic fibrosis, haemoglobinopathies)^36,37^ and ECS.^7^ It is important that future research further explores the support and potential role of GPs in offering population-based ECS.

Interestingly, GPs and midwives thought that population-based ECS should ideally be provided in primary care to optimize equal access. As suggested by several study participants, public health or community services that are already involved in prevention and child health could also be considered as providers and might even be a better solution. To our knowledge, this has not been suggested in the literature on population-based ECS before, and these services have not been included in previous studies. To explore the desirability and feasibility of public health services providing population-based ECS, pilot studies on responsible implementation of population-based ECS involving these services are needed. Research should also take into account a possible tension between the prevention-aimed character of these services and the stated main aim of an ECS offer.

### Limitations

This study had some limitations. First, recruitment was restricted to the Netherlands. As the healthcare systems in other countries might be differently organized, particularly regarding the role of primary care and the role of community midwifery in the Dutch healthcare system, our findings may not be generalizable to other countries. In particular, attitudes towards population-based ECS in people with lower socioeconomic status or in less developed healthcare systems were not part of our topic list for the focus groups, and therefore we did not elaborate on this. Second, our sample of participants was a convenience sampling due to COVID-19, and we therefore primarily used networks in the Northern part of the Netherlands to recruit participants. In the Netherlands, uptake of prenatal screening is for example lower in northern provinces, which may also partly be a consequence of counsellors’ framing of the offer.^38^ Study participation and findings may therefore not be representative for GPs and midwives in all regions in the Netherlands. Third, the educational session on haemoglobinopathies and the Dutch carrier screening guideline for recessive diseases in high-risk groups prior to the focus groups intended to stimulate participation and inform participants about carrier screening, but might have affected the views of participants on population-based ECS versus a high-risk offer only.

## Conclusion

This focus group study on views of Dutch GPs and midwives towards population-based ECS indicates that, while participants believed that ECS could enhance reproductive autonomy and may therefore prevent suffering, many GPs and midwives questioned the desirability of such an offer due to negative ethical, societal, and/or psychosocial concerns. If implemented, most participants agreed that it would be feasible to implement population-based ECS, once certain requirements regarding training, reimbursement, and the time needed for adequate pretest counselling are met. They preferred it to be offered preconceptionally but also prenatally in primary care or within existing public health services. If the decision is made to implement population-based ECS, our findings suggest that primary care and public health services can be considered as a preferred setting for offering ECS. A more extensive, quantitative exploration of primary care professionals’ views on population-based ECS and their perceived involvement in the offer might be insightful, as support and answering the needs of future providers is essential. Furthermore, pilot studies assessing population-based ECS offers incorporating these potential primary care and public health providers may contribute to our knowledge on whether and how to organize population-based ECS in a responsible manner.

## Supplementary material

Supplementary material is available at *Family Practice* online.

cmad011_suppl_Supplementary_Material_S1

cmad011_suppl_Supplementary_Material_S2

## Data Availability

Data sharing is not applicable to this article as only qualitative data were generated and analysed in this study.

## References

[1] ACOG Committee Opinion No. 690: carrier screening in the age of genomic medicine. Obstet Gynecol. 2017;129(3):e35–e40. https://pubmed.ncbi.nlm.nih.gov/28225420/. 28225425 10.1097/AOG.0000000000001951

[2] Antonarakis SE. Carrier screening for recessive disorders. Nat Rev Genet. 2019;20(9):549–561. 31142809 10.1038/s41576-019-0134-2

[3] Solomon BD , Nguyen AD, Bear KA, Wolfsberg TG. Clinical genomic database. Proc Natl Acad Sci USA. 2013;110(24):9851–9855. 23696674 10.1073/pnas.1302575110 PMC3683745

[4] Fridman H , Yntema HG, Magi R, Andreson R, Metspalu A, Mezzavila M, Tyler-Smith C, Xue Y, Carmi S, Levy-Lahad E, et al. The landscape of autosomal-recessive pathogenic variants in European populations reveals phenotype-specific effects. Am J Hum Genet. 2021;108(4):608–619. 33740458 10.1016/j.ajhg.2021.03.004 PMC8059335

[5] Henneman L , Borry P, Chokoshvili D, Cornel MC, van El CG, Forzano F, Hall A, Howard HC, Janssens S, Kayserili H, et al. Responsible implementation of expanded carrier screening. Eur J Hum Genet. 2016;24(6):e1–e12. 10.1038/ejhg.2015.271 PMC4867464 26980105

[6] Lazarin GA , Haque IS. Expanded carrier screening: a review of early implementation and literature. Semin Perinatol. 2016;40(1):29–34. 26718446 10.1053/j.semperi.2015.11.005

[7] Schuurmans J , Birnie E, van den Heuvel LM, Plantinga M, Lucassen A, van der Kolk DM, Abbott KM, Ranchor AV, Diemers AD, van Langen IM. Feasibility of couple-based expanded carrier screening offered by general practitioners. Eur J Hum Genet. 2019;27(5):691–700. 30742054 10.1038/s41431-019-0351-3 PMC6462008

[8] van Dijke I , Lakeman P, Sabiri N, Rusticus H, Ottenheim CPE, Mathijssen IB, Cornel MC, Henneman L et al. Couples’ experiences with expanded carrier screening: evaluation of a university hospital screening offer. Eur J Hum Genet. 2021;29:1252–1258. 34155360 10.1038/s41431-021-00923-9 PMC8384865

[9] Ong R , Howting D, Rea A, Christian H, Charman P, Molster C, Ravenscroft G, Laing NG. Measuring the impact of genetic knowledge on intentions and attitudes of the community towards expanded preconception carrier screening. J Med Genet. 2018;55(11):744–752. 30068663 10.1136/jmedgenet-2018-105362

[10] Dive L , Newson AJ. Ethical issues in reproductive genetic carrier screening. Med J Aust. 2021;214(4):165–167.e1. 32981044 10.5694/mja2.50789 PMC7983899

[11] Best S , Long J, Theodorou T, Hatem S, Lake R, Archibald A, Freeman L, Braithwaite J. Health practitioners’ perceptions of the barriers and enablers to the implementation of reproductive genetic carrier screening: a systematic review. Prenat Diagn. 2021;41(6):708–719. 33533079 10.1002/pd.5914 PMC8252081

[12] Plantinga M , Birnie E, Abbott KM, Sinke RJ, Lucassen AM, Schuurmans J, Kaplan S, Verkerk MA, Ranchor AV, van Langen IM. Population-based preconception carrier screening: how potential users from the general population view a test for 50 serious diseases. Eur J Hum Genet. 2016;24(10):1417–1423. 27165008 10.1038/ejhg.2016.43 PMC5027688

[13] Kraft SA , Duenas D, Wilfond BS, Goddard KAB. The evolving landscape of expanded carrier screening: challenges and opportunities. Genet Med. 2019;21(4):790–797. 30245516 10.1038/s41436-018-0273-4 PMC6752283

[14] van den Heuvel LM , van den Berg N, Janssens A, Birnie E, Henneman L, Dondorp WJ, Plantinga M, van Langen IM et al. Societal implications of expanded universal carrier screening: a scoping review. Eur J Hum Genet. 2022:1–18. 10.1038/s41431-022-01178-8 PMC9822904 36097155

[15] Jans SM , Henneman L, de Jonge A, van El CG, van Tuyl LH, Cornel MC, Lagro-Janssen ALM. ‘A morass of considerations’: exploring attitudes towards ethnicity-based haemoglobinopathy-carrier screening in primary care. Fam Pract. 2013;30(5):604–610. 23629736 10.1093/fampra/cmt019 PMC3782062

[16] Janssens S , De Paepe A, Borry P. Attitudes of health care professionals toward carrier screening for cystic fibrosis. A review of the literature. J Community Genet. 2014;5(1):13–29. 23275180 10.1007/s12687-012-0131-z PMC3890063

[17] Metcalfe SA. Carrier screening in preconception consultation in primary care. J Community Genet. 2012;3(3):193–203. 22183783 10.1007/s12687-011-0071-z PMC3419291

[18] Benn P , Chapman AR, Erickson K, Defrancesco MS, Wilkins-Haug L, Egan JF, Schulkin J. Obstetricians and gynecologists’ practice and opinions of expanded carrier testing and noninvasive prenatal testing. Prenat Diagn. 2014;34(2):145–152. 24222397 10.1002/pd.4272

[19] Briggs A , Nouri PK, Galloway M, O’Leary K, Pereira N, Lindheim SR. Expanded carrier screening: a current survey of physician utilization and attitudes. J Assist Reprod Genet. 2018;35(9):1631–1640. 30069849 10.1007/s10815-018-1272-8 PMC6133809

[20] Cho D , McGowan ML, Metcalfe J, Sharp RR. Expanded carrier screening in reproductive healthcare: perspectives from genetics professionals. Hum Reprod. 2013;28(6):1725–1730. 23589535 10.1093/humrep/det091 PMC3657126

[21] Janssens S , Chokoshvili D, Vears D, De Paepe A, Borry P. Attitudes of European geneticists regarding expanded carrier screening. J Obstet Gynecol Neonatal Nurs. 2017;46(1):63–71. 10.1016/j.jogn.2016.08.012 27875676

[22] Lazarin GA , Detweiler S, Nazareth SB, Ashkinadze E. Genetic counselors’ perspectives and practices regarding expanded carrier screening after initial clinical availability. J Genet Couns. 2016;25(2):395–404. 26354338 10.1007/s10897-015-9881-1 PMC4799270

[23] Ready K , Haque IS, Srinivasan BS, Marshall JR. Knowledge and attitudes regarding expanded genetic carrier screening among women’s healthcare providers. Fertil Steril. 2012;97(2):407–413. 22137493 10.1016/j.fertnstert.2011.11.007

[24] van der Hout S , Holtkamp KC, Henneman L, de Wert G, Dondorp WJ. Advantages of expanded universal carrier screening: what is at stake? Eur J Hum Genet. 2016;25(1):17–21. 27677414 10.1038/ejhg.2016.125 PMC5159761

[25] Delatycki MB , Alkuraya F, Archibald A, Castellani C, Cornel M, Grody WW, Henneman L, Ioannides AS, Kirk E, Laing N, et al. International perspectives on the implementation of reproductive carrier screening. Prenat Diagn. 2020;40(3):301–310. 31774570 10.1002/pd.5611

[26] M’Hamdi HI , van Voorst SF, Pinxten W, Hilhorst MT, Steegers EA. Barriers in the uptake and delivery of preconception care: exploring the views of care providers. Matern Child Health J. 2017;21(1):21–28. 27423236 10.1007/s10995-016-2089-7 PMC5226984

[27] Chun Tie Y , Birks M, Francis K. Grounded theory research: a design framework for novice researchers. SAGE Open Med. 2019;7:2050312118822927. 30637106 10.1177/2050312118822927 PMC6318722

[28] LaForge KG , Stack E, Livingston CJ, Hildebran C. Using asynchronous online focus groups to capture healthcare professional opinions. Int J Qual Methods. 2022;21.

[29] Braun V , Clarke V. Using thematic analysis in psychology. Qual Res Psychol. 2006;3(2):77–101.

[30] Holtkamp KC , Vos EM, Rigter T, Lakeman P, Henneman L, Cornel MC. Stakeholder perspectives on the implementation of genetic carrier screening in a changing landscape. BMC Health Serv Res. 2017;17(1). 10.1186/s12913-017-2083-9 PMC5314610 28209157

[31] van der Hout S , Dondorp W, de Wert G. The aims of expanded universal carrier screening: autonomy, prevention, and responsible parenthood. Bioethics. 2019;33(5):568–576. 30734373 10.1111/bioe.12555 PMC6594088

[32] Edwards JG , Feldman G, Goldberg J, Gregg AR, Norton ME, Rose NC, Schneider A, Stoll K, Wapner R, Watson MS. Expanded carrier screening in reproductive medicine-points to consider: a joint statement of the American College of Medical Genetics and Genomics, American College of Obstetricians and Gynecologists, National Society of Genetic Counselors, Perinatal Quality Foundation, and Society for Maternal-Fetal Medicine. Obstet Gynecol. 2015;125(3):653–662. 25730230 10.1097/AOG.0000000000000666

[33] Rothwell E , Johnson E, Mathiesen A, Golden K, Metcalf A, Rose NC, Botkin JR. Experiences among women with positive prenatal expanded carrier screening results. J Genet Couns. 2017;26(4):690–696. 27796679 10.1007/s10897-016-0037-8 PMC5432405

[34] De Wert GM , Dondorp WJ, Knoppers BM. Preconception care and genetic risk: ethical issues. J Community Genet. 2012;3(3):221–228. 22205578 10.1007/s12687-011-0074-9 PMC3419287

[35] Nijmeijer SCM , Conijn T, Lakeman P, Henneman L, Wijburg FA, Haverman L. Attitudes of the general population towards preconception expanded carrier screening for autosomal recessive disorders including inborn errors of metabolism. Mol Genet Metab. 2019;126(1):14–22. 30563741 10.1016/j.ymgme.2018.12.004

[36] Henneman L , Bramsen I, van Kempen L, van Acker MB, Pals G, van der Horst HE, Adèr HJ, van der Ploeg HM, ten Kate LP. Offering preconceptional cystic fibrosis carrier couple screening in the absence of established preconceptional care services. Community Genet. 2003;6(1):5–13. 12748433 10.1159/000069540

[37] Lakeman P , Plass AM, Henneman L, Bezemer PD, Cornel MC, ten Kate LP. Preconceptional ancestry-based carrier couple screening for cystic fibrosis and haemoglobinopathies: what determines the intention to participate or not and actual participation? Eur J Hum Genet. 2009;17(8):999–1009. 19223934 10.1038/ejhg.2009.1 PMC2986548

[38] van der Meij KRM , de Groot-van Mooren M, Carbo EWS, Pieters MJ, Rodenburg W, Sistermans EA, Cornel MC, Henneman L; Dutch NIPT Consortium. Uptake of fetal aneuploidy screening after the introduction of the non-invasive prenatal test: a national population-based register study. Acta Obstet Gynecol Scand. 2021;100(7):1265–1272. 33465829 10.1111/aogs.14091 PMC8359325

