# Supplementary material for: Primary care professionals’ views on population-based expanded carrier screening: an online focus group study
Source: Fam Pract. 2023 Feb 1;41(4):571–8. doi: 10.1093/fampra/cmad011 (PMC11324326; doi:10.1093/fampra/cmad011)
Supplement: cmad011_suppl_Supplementary_Material_S1 [file cmad011_suppl_supplementary_material_s1.docx]

**SUPPLEMENTARY MATERIAL S1**

**Primary care professionals’ views on population-based expanded carrier screening: an online focus group study – Lieke M. van den Heuvel, Anke J. Woudstra, Sanne van der Hout, Suze Jans, Tjerk Wiersma, Wybo Dondorp, Erwin Birnie, Phillis Lakeman, Lidewij Henneman, Mirjam Plantinga, Irene M. van Langen**

**List of networks approached**

| **Organisation** | **Description** | **Participated** |
| --- | --- | --- |
| Royal Dutch Organisation of Midwives (KNOV) | Professional organisation of and for midwives | Yes |
| Consortium Pregnancy and Birth Northern Netherlands (ZeGGN) | Knowledge network of community midwive practices in the three northern provinces of the Netherlands (Drenthe, Friesland, and Groningen) | Yes |
| GP network Maastricht UMC+ | Network of GPs working in the southern part of the Netherlands | No |
| Academic Network Family Medicine (AHN) | Department of Family Medicine Amsterdam UMC, which collaborates with GP practices in the region of Amsterdam | Yes |
| Department of family medicine University Medical Centre Groningen | Department of Family Medicine University Medical Centre Groningen, teaching GPs in training | Yes |
| Midwifery Academy Amsterdam Groningen (AVAG) | Education and research institute focused on midwifery | Yes |
