# Supplementary material for: Primary care professionals’ views on population-based expanded carrier screening: an online focus group study
Source: Fam Pract. 2023 Feb 1;41(4):571–8. doi: 10.1093/fampra/cmad011 (PMC11324326; doi:10.1093/fampra/cmad011)
Supplement: cmad011_suppl_Supplementary_Material_S2 [file cmad011_suppl_supplementary_material_s2.docx]

**SUPPLEMENTARY MATERIAL S2**

**Primary care professionals’ views on population-based expanded carrier screening: an online focus group study – Lieke M. van den Heuvel, Anke J. Woudstra, Sanne van der Hout, Suze Jans, Tjerk Wiersma, Wybo Dondorp, Erwin Birnie, Phillis Lakeman, Lidewij Henneman, Mirjam Plantinga, Irene M. van Langen**

**Topic list online focus groups midwives and general practitioners (GPs)**

| **Items** | **To be discussed** |
| --- | --- |
| **INTRODUCTION** | - Introducing each participant - Explanation of purpose and structure of focus group |
| **CARRIER SCREENING IN GENERAL** | - Attitude towards expanded carrier screening (ECS)   - Desirability - Experience with offering carrier tests |
| **ETHICAL ASPECTS OF ECS AND VIEWS ON THESE ASPECTS** | - Ethical aspects   - Purpose of ECS   - Panel of conditions   - Autonomy   - Consequences for relationship with partner   - Responsibilities   - Consequences for parents/family with a (child) with a hereditary condition   - Privacy   - Timing of offer |
| **PRECONCEPTIONAL SCREENING AS PART OF SCREENING CHAIN** | - Attitude towards consequences of carrier screening for preconception, prenatal and neonatal setting - Attitude towards optimal and timing of offer (preconception, prenatal, neonatal) - Expected effect on other reproductive screening offers (prenatal/neonatal) and views on this |
| **IMPLEMENTATION OF ECS - OFFER** | - Attitude towards method of offering ECS and results - Attitude towards funding and reimbursement (also regarding accessibility) - Ideas on information provision to individuals/couples (also in relation to the aim of ECS) |
| **IMPLEMENTATION OF ECS – PREREQUISITES** | - Perceived role and desired responsibility of midwives/GPs in offering screening - Views on ECS as part of preconception care - Views on required experience/expertise with offering ECS - Perceived prerequisites/needs for training, information provision and support |

**Introduction**

Welcome to this online focus group. My name is Lieke van den Heuvel and I will act as moderator in this group. Thank you for participating. In this group discussion, I would like to discuss your professional opinion regarding the desirability and feasibility of offering expanded carrier screening to couples with a desire to have children, and the preconditions for its implementation.

**BACKGROUND INFORMATION 1:** Carrier screening involves autosomal recessive or x-linked disorders. It is technically possible to test for dozens to more than hundreds of recessive and x-linked conditions simultaneously. This is called expanded carrier screening (https://www.huisartsengenetica.nl/info/dragerschapsscreening). With autosomal recessive conditions, if both parents are carriers, they have a 1 in 4 chance of having a child with the disease in each pregnancy, such as with Cystic Fibrosis. In x-linked disorders, there is an increased risk of the disorder if the female partner is a carrier of the genetic predisposition, as in Duchenne disease. In autosomal recessive and also many x-linked disorders, it is often unknown that people are carriers, because it has not shown up in the family before. Couples who know they are a carrier couple can make informed choices about having children, including using an egg or sperm donor, using preimplantation genetic diagnosis (embryo selection via In Vitro Fertilisation (IVF)) or becoming pregnant naturally and opting for prenatal diagnosis.

Asynchronous: In this group discussion, every day from Monday to Friday, I will upload one or more questions on the forum to which I would like you to respond. I would also like to ask you to respond to each other’s responses. On the last days no new questions will be posted; you will have the opportunity on those days to respond to earlier questions.

Synchronous: In this group discussion, all participants are currently logged in. The group discussion will last approximately 1.5 hours. I will ask you questions during the group discussion. I will also ask you to respond to each other.

**CARRIER SCREENING GENERAL (ASYNCHRONOUS: DAY 1)**

The first questions are about your opinion regarding your experiences with carrier screening for recessive conditions for couples with a desire to have children and your opinion about this (https://www.huisartsengenetica.nl/info/dragerschapsscreening). I may ask additional questions based on your response. I would like to ask you to answer these questions as well.

**Question 1:** Do you have experience with offering a carrier status test to couples who want to have children? If so, what are your experiences?

*Sub-questions (where possible introduced by moderator during discussion):*

1. Do you have specific experience with:
   1. Referring for a carrier test because of a positive family anamnesis, such as an autosomal recessive or X-linked condition in the family (e.g., cystic fibrosis)? If so, to whom did you refer and what was your experience with it?
   2. Offering a (preconception) carrier test yourself?
   3. Requesting a (biochemical) carrier test for haemoglobinopathies, such as sickle cell disease or thalassemia?

**BACKGROUND INFORMATION 2:** At the moment, carrier tests are actively offered to high-risk groups (for example couples who are at increased risk due to their ethnic origin; for more information, see the recent guideline 'Preconception Carrier Testing (PDO) for High-Risk Groups'). With broad preconception carrier screening, a carrier test for several conditions would be offered simultaneously to all couples with a possible desire to have children, regardless of their ethnic origin. Couples have a 1 in 100-150 chance of being carriers.

Question 2: What do you think of offering ECS (i.e., the provision of preconception screening information) to all couples who want to have children instead of only to high-risk couples?

*Sub-questions (where possible introduced by moderator during discussion):*

1. Do you think there is a need for such an offer to this target group?
2. Do you consider ECS for all couples who want to have children as necessary care or perhaps as a luxury product?
3. Can you think of possible consequences of receiving an offer and a test result for such a carrier test for the individual/couple and for a possible future child (both positive and negative)?
4. Can you think of possible consequences of offering screening to all couples who want to have children for society (both positive and negative)?
5. Can you think of possible consequences of offering a test to all couples with a desire to have children for health care providers (both positive and negative)?

**ETHICAL ASPECTS & CONSEQUENCES OF CARRIER SCREENING (ASYNCHRONOUS: DAY 2)**

The following questions focus on the possible ethical implications and societal impact of offering ECS to all couples who wish to have children.

Question 3: What would you consider the most important goal of offering carrier screening to all couples who want to have children? And what objections do you see to this? Do you also see other aims? What do you think of them?

*Sub questions (where possible introduced by moderator during discussion):*

1. What types of disorders should be included in such a supply?
   1. E.g., only 'serious' conditions? What do you understand by 'serious'?
      1. Should conditions occurring later in life also be included?
      2. Should conditions with treatable symptoms also be included?
      3. Should conditions with variable manifestations be included?
      4. Should the couple be able to make their own choice regarding the type of conditions that will result in a result in case of joint carrier?
2. How do you view (for example):
   1. Preventing suffering/'prevention'?
      1. What suffering do you think could be prevented with ECS?
      2. What responsibility do you see for yourself as a GP/midwife in this context in the prevention of suffering?
      3. Do you think that future parents have a responsibility to use ECS if they can prevent suffering in their children?
      4. Do you think that offering ECS would be a 'strive for a perfect society'?
   2. Promoting autonomous and informed decisions regarding reproductive options?
      1. In your opinion, would offering ECS to all couples wishing to have children contribute to this?
         1. If so, in what way?
         2. If not, why not?
   3. Reducing healthcare costs
      1. Do you think that preventing the birth of children with a high healthcare cost profile should also be a goal of offering ECS?

Question 4: Do you see any other ethical and/or societal aspects that should be considered in the decision whether or not to offer ECS to all couples who want to have children? If so, which? E.g., in terms of individual choices, for people with a disease, healthcare, costs of such tests, consequences for society, etc. (to prime later on)

*Sub-questions (where possible introduced by moderator during discussion):*

1. How do you view (for example):
   1. ECS and making autonomous (informed) choices about relationships, wanting children and pregnancy?
   2. The possibility that couples are seen or held responsible if they make certain choices as a result of ECS?
   3. ECS and a possible moral responsibility for couples to undergo such a test?
   4. The responsibility of healthcare providers to inform future parents of the possibility to be tested for carrier status?
   5. ECS and possible consequences for the care of people with conditions that are tested for in a carrier test?
   6. ECS and equal access to care? (in terms of cost, knowledge, culture, education, etc.)
   7. ECS and the medicalisation of childbearing and pregnancy?
   8. ECS and striving to a 'perfect society' and/or the 'perfect child'?
   9. The various reproductive options that carrier screening would allow for?
   10. ECS and trust in the general practitioner/ midwife?
   11. ECS and privacy of couples wanting to have children?

**CARRIER SCREENING PRECONCEPTION PREGNANCY SCREENING AS PART OF THE SCREENING CHAIN (ASYNCHRONOUS: DAY 3)**

The following questions are about your vision on pre-conception carrier screening as part of the screening chain regarding childbearing, pregnancy, and birth.

Question 5: If ECS would be introduced, what, in your opinion, is the optimal moment for offering ECS (prior to pregnancy (preconception), prenatally, neonatally, or already in young adulthood as an individual)? How could (preconception) ECS complement current reproductive screening offers (prenatal/neonatal)?

*Sub-questions (where possible introduced by moderator during discussion):*

1. What characteristics or factors (such as age, level of education) of the person(s) to whom ECS would be offered or the potential consequences this would have (such as screening at a young age) play a role?
2. Should ECS also be offered prenatally or neonatally? Why or why not?
3. Looking at the different types of screening around pregnancy and birth (preconception, prenatal and neonatal), do you think they have the same or different aims?
4. In your opinion, is the provision of (preconception) ECS more or less morally sensitive than other types of screening around pregnancy and birth?

Question 6: Do you think that the offer of ECS to couples with a desire to have children will influence participation in other, already implemented tests around pregnancy and birth (prenatal: NIPT/echo & neonatal: heel prick and hearing test)?

*Sub questions (where possible introduced by moderator during discussion):*

1. If yes, what do you expect to be positive and/or negative consequences of this? What is your opinion on this?
2. If not, why not?

**IMPLEMENTATION CARRIER SCREENING - OFFER (ASYNCHRONOUS: DAY 4)**

The following questions deal with the offer of such a carrier test to couples who wish to have children, and how this should be structured in your opinion.

Question 7: If ECS is introduced: In what way could ECS for all couples with a desire to have children be offered best? E.g., offer by the government, offer to all couples or only if couples ask for it, a result for the individual or the couple and financing/compensation of the test?

*Sub questions (where possible introduced by moderator during discussion):*

1. What do you think should be the government's role in this (e.g., equal to or different from prenatal or neonatal screening)?
   1. Alternatives would be for couples to pay for it themselves, or for hospitals or commercial laboratories to offer it. What are your thoughts on this?
   2. Would a national offer regulated by the government be important to ensure quality of carrier screening? Can you explain your answer?
2. How do you think ECS should be financed?
   1. E.g., reimbursed by insurance, by the government, (partly) by the individual or couple themselves? And why?
   2. Can the argument that ECS can contribute to reducing costs of care for people with a disease or disability play a role in this? Why yes/no?

**IMPLEMENTATION OF ECS - ROLE OF MIDWIFE (ASYNCHRONOUS: DAY 5)**

The following questions are about how you view your own role in offering ECS and what, in your opinion, is needed to fulfil this role.

Question 8: Do you see a role for your professional group in the provision of ECS for couples wanting to have children? If so, which? If not, why not?

*Sub questions (where possible introduced by moderator during discussion):*

1. What do you think the offer by your discipline would look like?

**BACKGROUND INFORMATION 3:** Preconception care focuses on the identification of a possible increased risk of adverse pregnancy outcomes prior to pregnancy. In addition, it provides general and, where appropriate, targeted advice to promote health and, if necessary, to initiate timely treatment.

1. Could/should a test offer be part of the preconception care consultation?
2. Should every GP/midwife be able to offer ECS? Can you explain your answer?
3. Do you think other professional groups could (also) offer ECS? Would you consider this desirable?

Question 9: If your discipline were to offer ECS to all couples who wish to have children, what would be needed, in your opinion, to realise this offer and what would be potential barriers (e.g., information/training, financing, time, minimum number of pre-test consultations per year)?

*Sub questions (where possible introduced by moderator during discussion):*

1. Would you consider training desirable to offer ECS? If so, only at the start of the ECS offer or repeated training? What do you consider important elements of such training (knowledge/skills)? How should this be assessed?
2. How many times a year do you think you, as a midwife/GP, should counsel/confront couples in order to maintain sufficient expertise?
   1. How do you think this can be realised 'sustainably'?
